# Sortilin-Related Receptor SORCS3 Is a Postsynaptic Modulator of Synaptic Depression and Fear Extinction

**DOI:** 10.1371/journal.pone.0075006

**Published:** 2013-09-19

**Authors:** Tilman Breiderhoff, Gitte B. Christiansen, Lone T. Pallesen, Christian Vaegter, Anders Nykjaer, Mai Marie Holm, Simon Glerup, Thomas E. Willnow

**Affiliations:** 1 Max-Delbrueck-Center for Molecular Medicine, Berlin, Germany; 2 Department of Biomedicine, Aarhus University, Aarhus, Denmark; 3 The Lundbeck Foundation Research Centre MIND and the Danish Research Institute of Translational Neuroscience, Aarhus University, Aarhus, Denmark; Georgia Regents University, United States of America

## Abstract

SORCS3 is an orphan receptor of the VPS10P domain receptor family, a group of sorting and signaling receptors central to many pathways in control of neuronal viability and function. SORCS3 is highly expressed in the CA1 region of the hippocampus, but the relevance of this receptor for hippocampal activity remained absolutely unclear. Here, we show that SORCS3 localizes to the postsynaptic density and that loss of receptor activity in gene-targeted mice abrogates NMDA receptor-dependent and -independent forms of long-term depression (LTD). Consistent with a loss of synaptic retraction, SORCS3-deficient mice suffer from deficits in behavioral activities associated with hippocampal LTD, particularly from an accelerated extinction of fear memory. A possible molecular mechanism for SORCS3 in synaptic depression was suggested by targeted proteomics approaches that identified the ability of SORCS3 to functionally interact with PICK1, an adaptor that sorts glutamate receptors at the postsynapse. Faulty localization of PICK1 in SORCS3-deficient neurons argues for altered glutamate receptor trafficking as the cause of altered synaptic plasticity in the SORCS3-deficient mouse model. In conclusion, our studies have identified a novel function for VPS10P domain receptors in control of synaptic depression and suggest SORCS3 as a novel factor modulating aversive memory extinction.

## Introduction

VPS10P domain receptors are a unique class of sorting and signaling receptors expressed in the nervous system. Five receptors form this gene family in mammals designated sortilin [1], sorting-related receptor with A-type repeats (SORLA) [2,3] as well as SORCS1, SORCS2, and SORCS3 [4]. All family members are characterized by a 700 amino acid module in their extracellular domain, initially identified in the yeast sorting receptor VPS10P (vacuolar protein sorting 10 protein) [5]. In recent years, three VPS10P domain receptors have been studied in detail documenting the central role played by this gene family in control of neuronal viability and function (reviewed in 6). Thus, sortilin was shown to regulate neuronal cell death and survival through modulation of (pro)-neurotrophin signaling [7,8,9,10]. SORLA and SORCS1 act as neuronal receptors for the amyloid precursor protein (APP) controlling proteolytic breakdown of this precursor into neurotoxic amyloid-β peptides, a pathological mechanism in Alzheimer’s disease [11,12,13,14,15].

Contrary to other VPS10P domain receptors, the significance of SORCS3 for the nervous system is absolutely unclear. Together with SORCS1 and SORCS2, SORCS3 forms a closely related subgroup within the VPS10P domain receptor family characterized by the presence of one amino terminal VPS10P domain followed by an imperfect leucine-reach repeat in their extracellular regions [4]. SORCS3 is a 130 kDa neuronal orphan receptor distinctly expressed in the hippocampus and cortex, and to a lesser extend in the cerebellum [4,16]. Hippocampal expression in mice is markedly up-regulated by synaptic activity following induction of limbic seizures through kainic acid injection [16].

Here, we report the generation and functional characterization of a SORCS3-deficient mouse model to query the relevance of this receptor for hippocampal activity *in vivo*. SORCS3-deficient animals are viable and fertile, but exhibit profound alterations in synaptic plasticity as shown by loss of long-term depression (LTD). Altered synaptic plasticity coincides with defects in spatial learning and modulation of fear memory, indicating involvement of SORCS3 in anxiety-related hippocampal activities. The ability of the receptor to control the subcellular localization of PICK1, an adaptor that sorts glutamate receptors, suggests a molecular mechanism for SORCS3 in control of neurotransmitter receptor trafficking at the synapse.

## Materials and Methods

### Materials

A polyclonal antiserum against the extracellular portion of murine SORCS3 was produced in-house by immunizing rabbits with the recombinant protein fragment purified from transfected HEK293 cells. The antiserum was affinity purified using the recombinant antigen. Antisera directed against the following proteins were obtained from commercial suppliers: PDS95, GluR2, NR2B, mGluR5 (NeuroMab); synaptophysin (Synaptic Systems); GluR1, p75NTR, TrkB (Cell Signalling); PICK1 (Novus Biologicals, NeuroMab), Na^+^/K^+^ ATPase α-1 subunit (Merck Millipore Cat. 05-369), SORCS2 (A. Nykjaer, Aarhus).

### Hippocampal synaptosomal preparations

Hippocampi of mice were dissected and homogenized in 320 mM sucrose, 4 mM HEPES (pH 7.4), with 10 strokes of a glass-teflon homogenizer (H, homogenate). The homogenate was centrifuged at 1000xg for 10 min and the postnuclear supernatant (S1) was further centrifuged at 10,000xg for 15 min to yield the crude synaptosomal pellet (P2) and a supernatant, of which the light membrane fraction (P3) was pelleted at 150,000xg for 30 min. After washing the pellet, P2 was lysed by hypo-osmotic shock and three strokes of a glass-teflon homogenizer. The lysate was centrifuged at 25,000xg for 20 min to yield the lysed synaptosomal membrane fraction (LP1). The supernatant (LS1) was pelleted at 165,000xg for 2 hours to derive the crude synaptic vesicle fraction (LP2). LP1 was layered on top of a discontinuous gradient containing 0.8 to 1.0 to 1.2 M sucrose and centrifuged at 150,000xg for 2 hours. Synaptic plasma membranes (SPM) were recovered in the layer between 1.0 and 1.2 M sucrose, diluted to 0.32 M sucrose and centrifuged at 150,000xg for 30 min. After resuspension and addition of Triton X-100 (final concentration 0.5%), SPMs were incubated on a rotating wheel at 4°C for 15 min, followed by centrifugation at 32,000xg (20 min) to yielded the PSDI pellet. The pellet was resuspended in 0.5% Triton X100, incubated on a rotating wheel at 4°C for 15 min and centrifuged at 200,000xg (20 min) to obtain the PSDII pellet.

### Affinity purification and characterization of SORCS3 interacting proteins

Proteins were isolated using synthetic peptides corresponding to the carboxyl terminal oligopeptides of SORCS3 (S^1207^ESTKEIPNCTSV; UniProt Q8VI51) or SORCS2 (S^1147^INSREMHSYLVG; Q9EPR5). Peptides were coupled to sepharose beads and incubated with total brain lysate (containing 1% Triton X-100) at 4°C over night. Beads were pelleted and bound proteins eluted by boiling in Laemmli buffer. After separation on SDS-PAGE, protein bands were stained with Coomassie, cut out from the gel, and identified by mass spectrometry.

For co-immunoprecipitation, constructs encoding SORCS3 and CS3^CTSV^ were transiently transfected into COS-7 cells. After 36 hours, the cells were sonicated in 150 mM NaCl, 50 mM Tris (pH8), 1% NP-40 substitute, and 1 mM 0.25% deoxycholate, and incubated with 1µg of IgG directed against PSD95 at 4°C over night. Immunoprecipitates were collected using Protein G agarose (Roche) according to manufacturer’s protocols. Eluted Proteins were separated on SDS-PAGE and analyzed by Western blotting.

For pull-down studies, HEK293 cells stably expressing SORCS3 were lysed on ice in lysis buffer (150mM NaCl, 2 mM MgCl_2_, 0.1 mM EGTA, 2 mM CaCl_2_, 10 mM HEPES, 1% Triton X-100, pH 7.4) and centrifuged at 4°C. 200 µl of the supernatant were mixed with either 10 µg GST or 15 µg GST-PICK1, adjusted to a volume of 1 ml in lysis buffer (without Triton), and rotated overnight at 4°C. The following day, glutathione sepharose beads were added and rotated for 5 hours at 4°C. Beads were pelleted, washed four times in lysis buffer (modified to 0.4 M NaCl, without Triton X-100), eluted, and analyzed by SDS-PAGE.

### Generation of SORCS3-deficient mice

For homologous recombination in embryonic stem (ES) cells, a targeting vector was constructed as detailed in the result section. In brief, regions homologous to the murine *Sorcs3* locus were amplified by PCR from isogenic ES cell DNA. A neomycin cassette flanked by FRT and loxP sites was inserted 0.8 kb downstream stream of exon 1 and one loxP site was inserted 0.8 kb upstream of exon 3. After electroporation of the targeting construct into ES cells and selection with G418, clones with homologous recombination were identified by Southern blot analysis. Mice derived by standard blastocyst injection of targeted cell clones were bred to the Cre deleter strain [17] to remove the neomycin cassette and to derive animals heterozygous for the deleted *Sorcs3* allele (*Sorcs3* ^*+/-*^). *Sorcs3* ^*+/-*^ animals were backcrossed on C57Bl/6N for more than 10 generations and then bred to homozygosity for the deleted allele (*Sorcs3* ^*-/-*^). Experiments in this manuscript have been carried out in littermates including all behavioral and biochemical analyses and most of the electrophysiological studies. The only exception being the mGluR-dependent LTD study which was performed using separate C57Bl/6N inbred lines of wild type or *Sorcs3* null background. Animal experimentation was performed after approval by local ethics committees (x9012/12, LAGESO, Berlin, Germany; 2011/561-119, University of Aarhus, Aarhus, Denmark).

For RT-PCR analysis of *Sorcs3* transcription, hippocampi, cortices and cerebella from freshly sacrificed mice were homogenized in TRIzol Reagent (Life Technologies). RNA was isolated using RNeasy Mini Kit (Qiagen) and transcribed to cDNA using the High Capacity RNA-to-cDNA Kit (Life Technologies). Specific cDNAs were amplified using Taq Polymerase (New England Biolabs) and the following primer: *Sorcs3* Ex1 forward: GCGGGGACTCTTGGGCTACTG, *Sorcs3* Ex2 reverse: GGTGGCGCCATAATCTACTGAC; *Sorcs3* Ex13 forward: TCCTAGACTGGGGTGGTGCTC; *Sorcs3* Ex15 reverse: CAGCGCCGGCTGAAGATAGAC; 18S forward: GTCCCCCAACTTCTTAGAG, 18S reverse GACCTACGGAAACTTGTTAC.

### Electrophysiological recordings

Mice were anesthetized with isoflurane and decapitated. The brain was removed and transferred to an ice-cold artificial cerebrospinal fluid (ACSF) composed of 126 mM NaCl, 2.5 mM KCl, 1.25 mM NaH_2_PO_4_, 2.5 mM CaCl_2_, 1.3 mM MgCl_2_, 10 mM D-glucose, 26 mM NaHCO_3_ (osmolality 305-315 mosmol · kg^-1^) at pH 7.4. ACSF used in this study was bubbled with carbogen (5% CO_2_/ 95% O_2_). Coronal slices (400 µm) were cut on a Vibratome 3000 Plus and stored for at least 90 minutes in ACSF at room temperature before recording.

Coronal brain slices were placed in an interface recording chamber perfused with ACSF containing 0.5% albumin that was recycled (a total volume of 300 ml; flow rate 2 ml/min). Recording electrodes (resistance 10-20 MΩ) were pulled from borosilicate glass (outer diameter: 1.5 mm; inner diameter: 0.8 mm, King Precision Glass Inc.) and filled with ACSF. The stimulation electrode (Concentric Bipolar Electrode, CBARC75, FHC Inc.) was placed in the stratum radiatum in the CA1 of the hippocampus and Schaffer collaterals were stimulated using a Stimulus Isolator (A365, World Precision Instruments) and a Master8 (A.M.P.I.). The recording electrode was placed in the stratum radiatum and recordings were made using MultiClamp 700B (Axon instruments) and Digidata 1440A (Axon Instruments). Extracellular field recordings were performed at 33 ± 1°C. After obtaining a stable baseline, low-frequency or tetanic stimulations were applied. Stimulus intensity was adjusted to evoke fEPSP at 40-50% of maximum. NMDA receptor-dependent LTD was induced by a single pulse applied at a rate of 1 Hz for 20 min. mGluR-dependent LTD was induced by twin pulses separated by 50 ms applied at 1Hz for 18 min. We induced long-term potentiation (LTP) by two trains of high-frequency stimulation (100 Hz, 1 s, separated by 45 sec). Paired-pulse facilitation (PPF) was induced by twin pulses with interstimulus intervals of 25-300 ms. Where indicated, 10 µM of the GABA_B_ receptor agonist baclofen (Tocris) was supplied to the recording chamber during the entire PPF experiment.

### Behavioral studies

All behavioral experiments were carried out using 14-20 weeks old male mice between 9: 00 a.m. to 5: 00 p.m. The open field test was performed in chambers monitored by an automated video system (TSE). Mice were placed in the chambers for 90 min on three consecutive days. The distance traveled was analyzed in 5 min intervals.

The experimental setup for the Barnes maze paradigm of spatial learning consisted of a circular platform (92 cm in diameter) elevated 105 cm above the floor. The maze contains 20 equally spaced holes (5 cm in diameter; 7.5 cm distance between the individual holes). The centers of all holes were 4.5 cm away from the perimeter of the maze. A hidden escape box was placed under the target hole. To facilitate spatial orientation, visual cues were placed on the laboratory walls. On day 1, mice were allowed to freely explore the maze for 3 min and were subsequently placed in the escape box for 2 min. Mice were then placed in the center of the maze under a dark circular box for 10 sec to ensure random orientation at the beginning of the test. A fan was turned on as reinforcing stimulus during the test phase. Mice were tested for four consecutive days and their latency to enter the target hole was measured by video recording and analysis using the Any-Maze tracking software.

Extinction of fear memory was assessed using a Gemini Avoidance System which takes advantage of the natural preference of mice for the dark. The setup consists of a brightly lit room and a dark room separated by a guillotine door. On the training day, individual mice are placed in the bright room. When entering the dark room, the door closes and the mouse receives an electric foot shock (0.4 mA for 1 sec). Twenty-four hours later, the mice are returned to the bright room and the latency to enter the dark room is recorded as an indicator of memory of the shock. Extinction of fear was achieved by returning the animals once every 24 h into the setup and by monitoring their latency to cross into the dark room for a total of 5 consecutive days. To test remote fear memory, animals were shocked and than returned once into the setup after 16 days.

For statistical analysis of behavioral studies, each measure of learning (Barnes Maze) or extinction of fear memory was analyzed with a mixed model ANOVA using GraphPad prism with genotype as a between-subject factor and trial day as a within-subject factor. If the repeated measures analysis showed significant differences between genotypes, *post hoc* analyses were performed at individual time points using the two-tailed Student’s *t*-test for independent samples. Differences were considered statistically significant if p<0.05.

## Results

### SORCS3 is expressed in the postsynaptic density of hippocampal neurons

**Figure 1:**
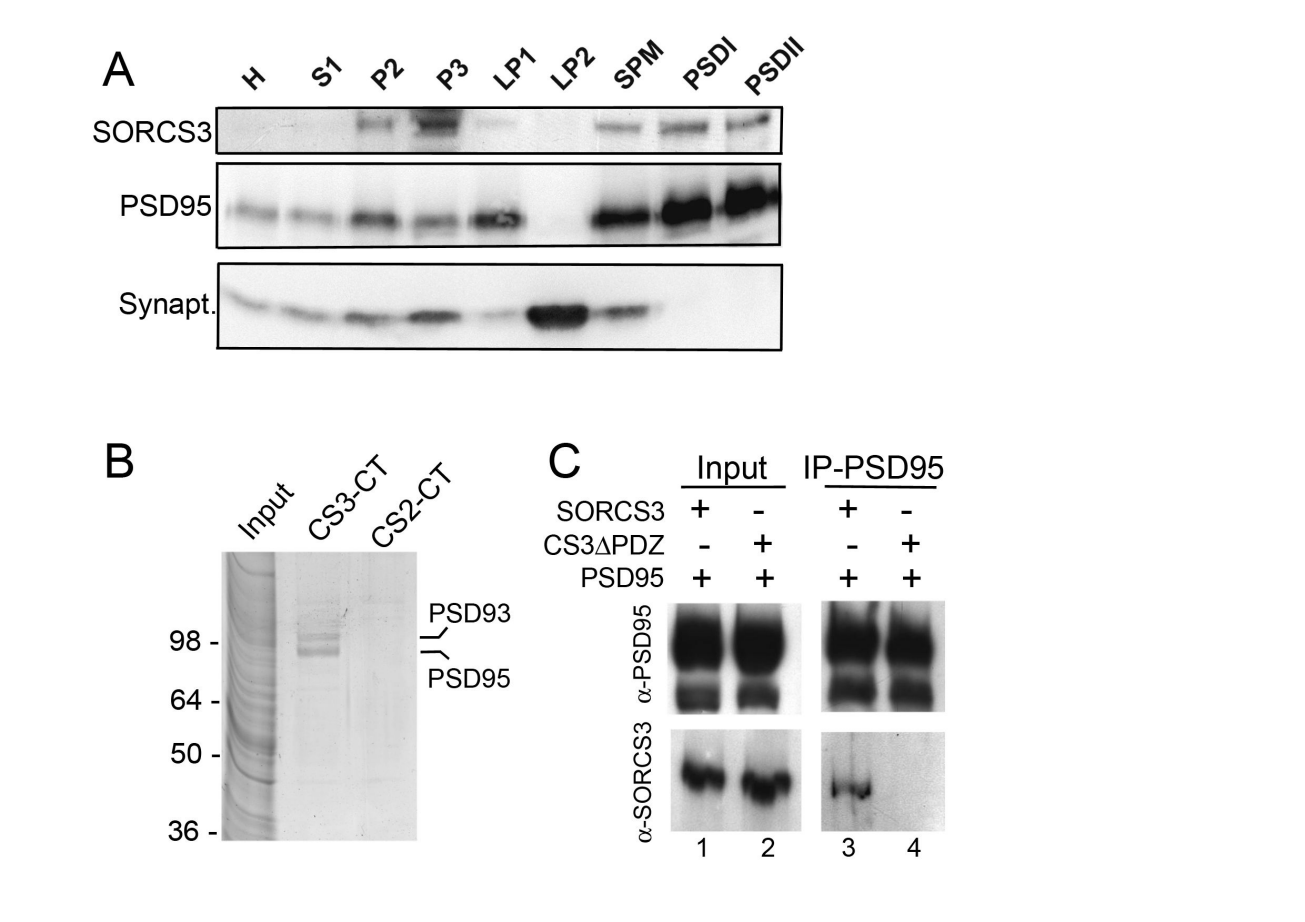
SORCS3 localizes to the post-synaptic density of hippocampal neurons. (**A**) Hippocampal extracts of wild types mice were subjected to subcellular fractionation as described in the method section. Western blot analysis identifies SORCS3 in crude synaptosomal membranes (P2), light membrane fraction (P3), synaptic plasma membranes (SPM), as well as in the post-synaptic densities (PSD) I and II. The receptor is not seen in the synaptic vesicle-enriched fraction (LP2). Detection of PSD95 and synaptophysin served as markers of post-synaptic densities and synaptic vesicles, respectively. H: Hippocampal homogenate. (**B**) Total mouse brain extracts were subjected to affinity purification on resin coupled with synthetic peptides encompassing 14 amino acids of the cytoplasmic tails of SORCS3 (CS3-CT) or SORCS2 (CS2-CT) as detailed in the method section. Two proteins purified from CS3-CT but not the CS2-CT column as shown by SDS-PAGE and staining with Coomassie. These proteins were identified as PSD93 and PSD95 by mass spectrometry. (**C**) COS7 cells were transiently transfected with constructs encoding PSD95 together with either full-length murine SORCS3 or a receptor variant lacking the PDZ domain binding site (CS3^CTSV^). SORCS3 (lane 3) but not CS3^CTSV^ (lane 4) co-immunoprecipitated with an anti-PSD95 antiserum (panel IP-PSD95). Panel Input (lanes 1 and 2) represents the cell lysate tested for PSD95 and SORCS3 prior to immunoprecipitation.

**Figure 2:**
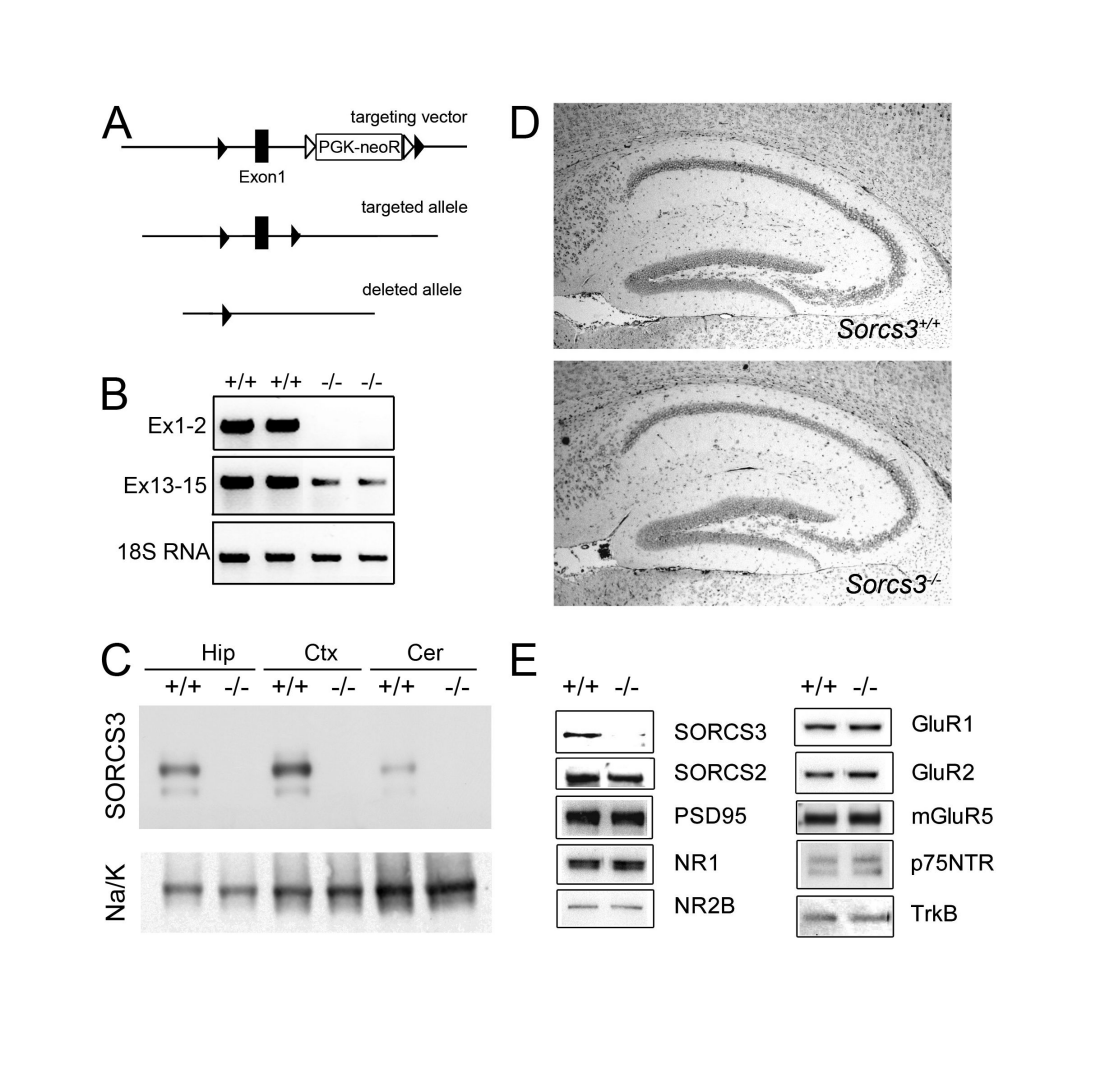
Generation and histological analysis of a SORCS3-deficient mouse model. (**A**) For targeted disruption of the murine *Sorcs3* locus, a targeting vector was constructed introducing the neomycin phosphotransferase gene driven by the phosphoglycerate kinase promoter (PGK-neoR) into intron 2 of the *Sorcs3* locus. The PGK-neoR cassette was flanked by FRT sites (open triangles). In addition, the vector introduced two loxP recombination sites (closed triangles) 5’ and 3’ of exon 1, respectively. Following standard homologous recombination in embryonic stem cells and blastocyst injections, mice carrying the modified gene through the germ line were bred with the flp deleter strain to remove PGK-neoR (targeted allele). For gene inactivation, mice were crossed with cre deleter mice to excise exon 1 that encodes the start codon and signal and pro-peptides of *Sorcs3* (deleted allele). (**B**) RT-PCR analysis on brain tissue documents complete loss of transcripts encoding exon 1 in *Sorcs3* ^*-/-*^ animals as compared to *Sorcs3* ^*+/+*^ controls. Minor amounts of an aberrant transcript encompassing exons 13-15 are seen. (C) Successful ablation of SORCS3 protein expression was confirmed by Western blot analysis of extracts from hippocampus (Hip), cortex (Ctx), and cerebellum (Cer) detecting SORCS3 in wild type mice (+/+), but in animals homozygous for the disrupted allele (-/-). Detection of Na^+^/K^+^ ATPase α-1 subunit (Na/K) served as loading control. (**D**) Histological sections stained with Nissl from hippocampi of *Sorcs3* ^*+/+*^ and *Sorcs3* ^*-/-*^ mice. (**E**) Western blot analysis of the indicated proteins in the PSD fraction of hippocampi from *Sorcs3* ^*+/+*^ and *Sorcs3* ^*-/-*^ mice. NR_1/2_B, NMDA glutamate receptor subunit 1/2B; GluR1/2, AMPA glutamate receptor subunit 1/2; mGlur5, metabotropic glutamate receptor type 5; p75NTR, nerve growth factor receptor; TrkB, neurotrophic tyrosine kinase, receptor, type 2.

Previously, in situ hybridization studies localized *Sorcs3* transcripts to the brain of mice. In the adult central nervous system, neuronal expression of *Sorcs3* is most pronounced in the CA1 region of the hippocampus. Additional patterns of expression are seen in the mitral cell layer of the olfactory bulb, in the piriform and cerebral cortex, as well as in the molecular layer of the cerebellum [16]. No significant expression of the gene in peripheral tissues has been reported thus far. Here, we used subcellular fractionation to substantiate expression of the receptor protein in the murine hippocampus. As shown in Figure 1A, SORCS3 was detected in fractions encompassing the synaptic plasma membrane or the postsynaptic density (PSD), but not in synaptic vesicle-enriched fractions. The localization of SORCS3 at the PSD was further supported by unbiased proteomics approaches to identify proteins that interact with the cytoplasmic tail of the receptor in neurons. To do so, synthetic peptides encompassing the last 14 amino acids of the cytoplasmic domain of SORCS3 (CS3-CT) were coupled to sepharose resin and used to affinity purify interacting proteins from mouse brain homogenates. Two prominent proteins were purified on CS3-CT columns that were identified as PSD93 and PSD95 by mass spectrometry (Figure 1B). These proteins were not recovered on columns containing a carboxyl terminal peptide of the related receptor SORCS2 (CS2-CT, Figure 2B). The ability of SORCS3 to interact with PSD95 was confirmed in transiently transfected COS7 cells showing co-immunoprecipitation of SORCS3 with an antiserum directed against PSD95 (Figure 1C). The interaction site for PSD95 was mapped to the last four amino acids of the cytosolic part of SORCS3 containing a putative PDZ domain binding motif (CTSV) as a receptor mutant lacking this tetrapeptide failed to co-immunoprecipitate with anti-PSD95 IgG (C3^CTSV^; Figure 1C). Although obtained *in vitro*, the above data provide experimental support for a potential interaction of SORCS3 with PSD95 *in vivo*.

### Generation of a SORCS3-deficient mouse model

To explore the relevance of SORCS3 for hippocampal activity, we next generated a mouse model carrying a targeted disruption of *Sorcs3*. To do so, loxP recombination sites flanking exon 1 of the *Sorcs3* locus on mouse chromosome 19D1 were introduced into the genome of mice using standard embryonic stem cell technology (Figure 2A). Exon 1 encodes 206 amino acid residues encompassing the Start-ATG, the signal peptide, and the propetide of SORCS3. Cre-mediated excision of this gene region resulted in complete elimination of *Sorcs3* transcripts encoding exon 1 (Figure 2B). Trace amounts of aberrant transcripts encompassing exons 13-15 were still produced from the targeted gene locus as shown by RT-PCR (Figure 2B). However, no wild type protein or any truncated receptor product were detected in hippocampus, cortex, or cerebellum of mice homozygous for the disrupted *Sorcs3* locus confirming successful inactivation of SORCS3 expression in our mouse model (Figure 2C).

Mice homozygous for the *Sorcs3* null allele (*Sorcs3* ^*-/-*^) were viable and fertile, and showed no discernable abnormalities upon external inspection. Also, histological analysis did not reveal any obvious histoanatomical alterations of the SORCS3-deficient hippocampus as compared to control tissue (Figure 2D). Finally, the level of expression of proteins in the PSD was not affected by lack of SORCS3 as shown in subcellular fractionation experiments for the related receptor SORCS2, for PSD95, or for subunits of AMPA receptors (GluR1, GluR2), NMDA receptors (NR1 and NR2B), or the metabotropic glutamate receptor mGluR5 (Figure 2E). Similarly, the levels of co-receptors to other VPS10P domain receptors such as the neurotrophin receptors P75^NTR^ and TrkB were unaltered (Figure 2E).

### SORCS3 deficiency results in loss of long-term depression

**Figure 3:**
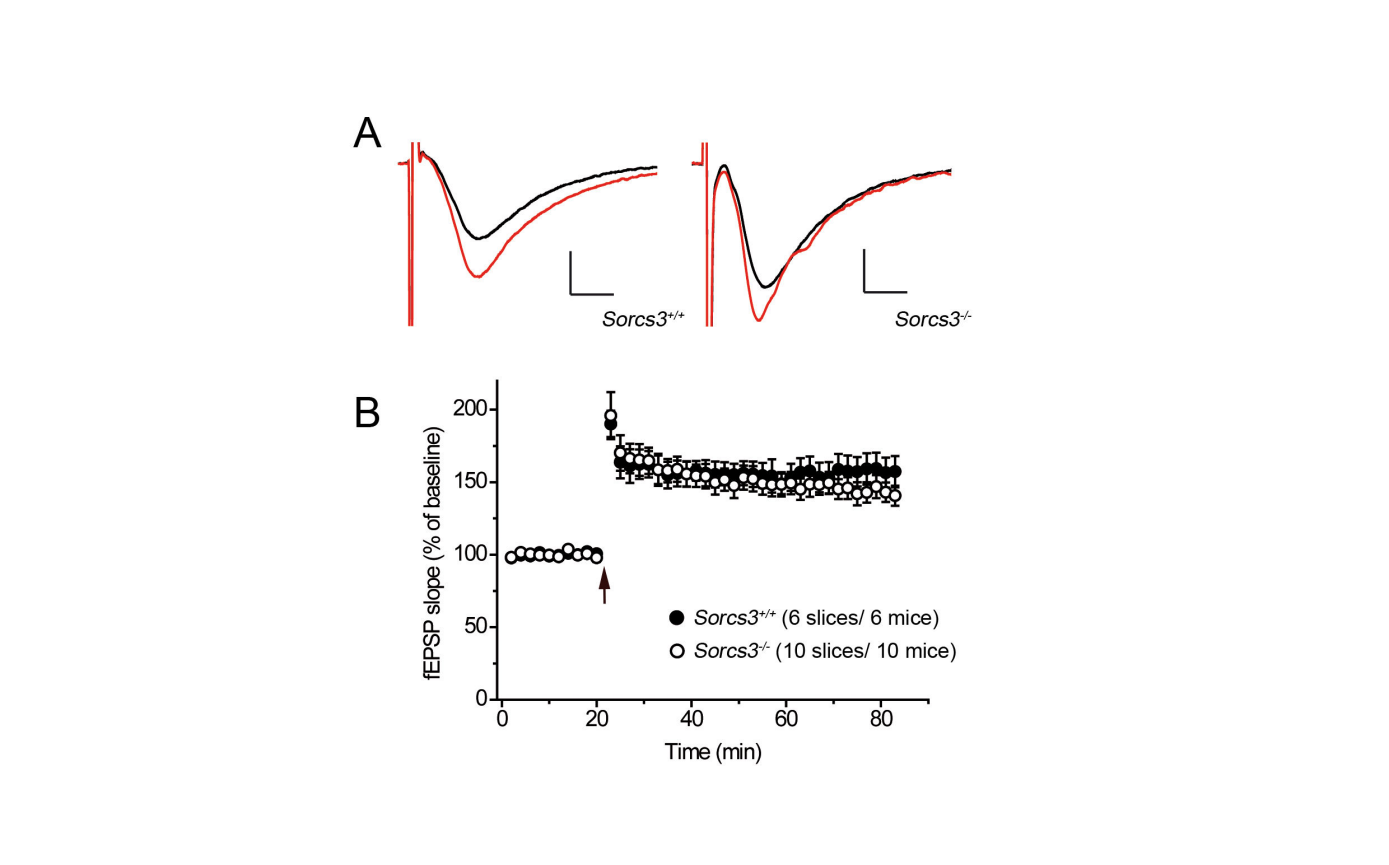
Long-term potentiation is normal in SORCS3-deficient mice. Field recordings in the stratum radiatum in the CA1 of the hippocampus after stimulation of Schaffer collaterals in slices of wild type and SORCS3-deficient mice. (**A**) Exemplary traces depicting fEPSPs before (black line) and after tetanic stimulation (red line) using a high-frequency 100 Hz protocol in slices from wild type and SORCS3-deficient mice. Scale bar: 0.4 mV/4 ms. (**B**) Averaged slopes of fEPSPs in wild type and in SORCS3-deficient slices before and after high-frequency stimulation (arrow). Data are given as mean ± SEM (+/+: 157.5 ± 11.0%; -/-: 142.5 ± 8.7%, p>0.05).

Given the prominent expression of SORCS3 in the CA1 region of the hippocampus, we performed electrophysiological recordings to explore the consequences of receptor deficiency for synaptic plasticity in this brain area. Long-term potentiation (LTP) is a major form of long-lasting synaptic plasticity [18]. During LTP, AMPA receptors are inserted into the PSD, resulting in an increase in synaptic strength [19,20]. However, field recordings of the stratum radiatum in the CA1 region of the hippocampus following stimulation of Schaffer collaterals failed to reveal any difference in early LTP in slices from SORCS3-deficient compared with wild type brains (Figure 3).

**Figure 4:**
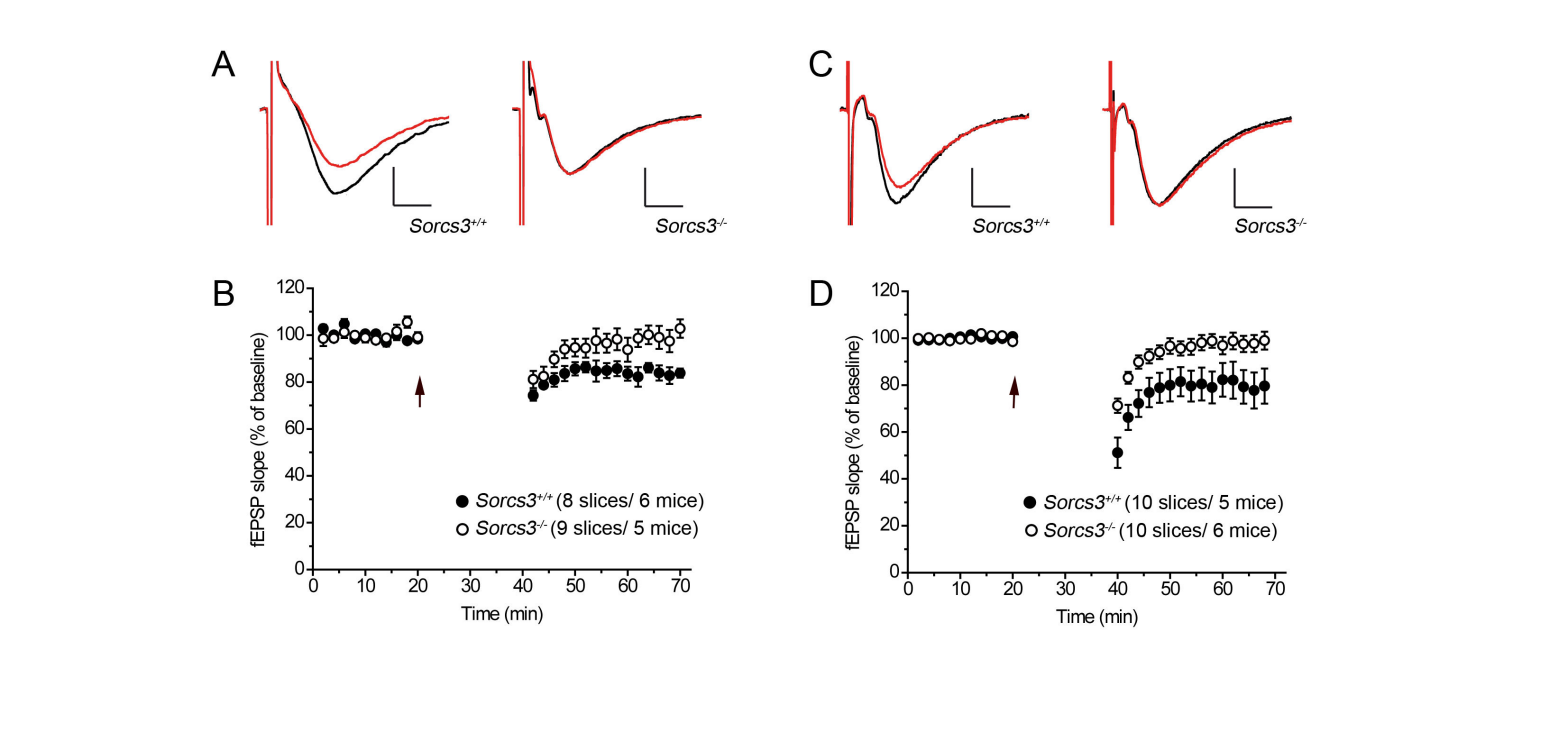
Long-term depression is impaired in SORCS3-deficient mice. Field recordings of the stratum radiatum in the CA1 of the hippocampus after stimulation of Schaffer collaterals in slices of wild-type and in SORCS3-deficient mice (**A**-**B**) A low-frequency 1 Hz protocol was applied to induce NMDA receptor-dependent long-term depression (LTD). (**A**) Representative traces depicting fEPSPs before (black line) and after low-frequency stimulation (red line) in wild type and receptor-deficient mice. (**B**) Averaged fEPSP slopes before and after low-frequency stimulation (arrow) in slices of wild type and SORCS3 deficient animals (+/+: 83.6 ± 4.2%; -/-: 99.2 ± 4.9%, p<0.05). (**C**-**D**) A low-frequency 1 Hz paired-pulse protocol was used to elicit mGluR-dependent LTD. (**C**) Exemplary traces depicting fEPSPs before (black line) and after low-frequency paired-pulse stimulation (red line) in wild type and receptor-deficient mice. (**D**) Averaged fEPSP slopes before and after low-frequency paired-pulse stimulation (arrow) are given (+/+: 78.3 ± 8.3%; -/-: 98.1 ± 4.4%, p < 0.05). Scale bars in A and C: 0.4 mV/4 ms. Data in B and D are given as mean ± SEM.

Long-term depression (LTD) is the second major form of long-lasting synaptic plasticity and considered important for synaptic retraction. During LTD, AMPA receptors undergo internalization from the PSD causing a decrease in synaptic strength. The most widely studied form of LTD is NMDA receptor-dependent. Here, the synaptic activation of NMDA receptors triggers the induction of LTD [19,21]. As shown in Figure 4A and B, NMDA receptor-dependent-LTD was absent in hippocampal slices from *Sorcs3* ^*-/-*^ animals. Another form of LTD is mGluR-dependent whereby synaptic activation of metabotropic glutamate receptors triggers LTD [19,22]. Remarkably, mGluR-dependent LTD was also lost in SORCS3-deficient mice (Figure 4C and D).

**Figure 5:**
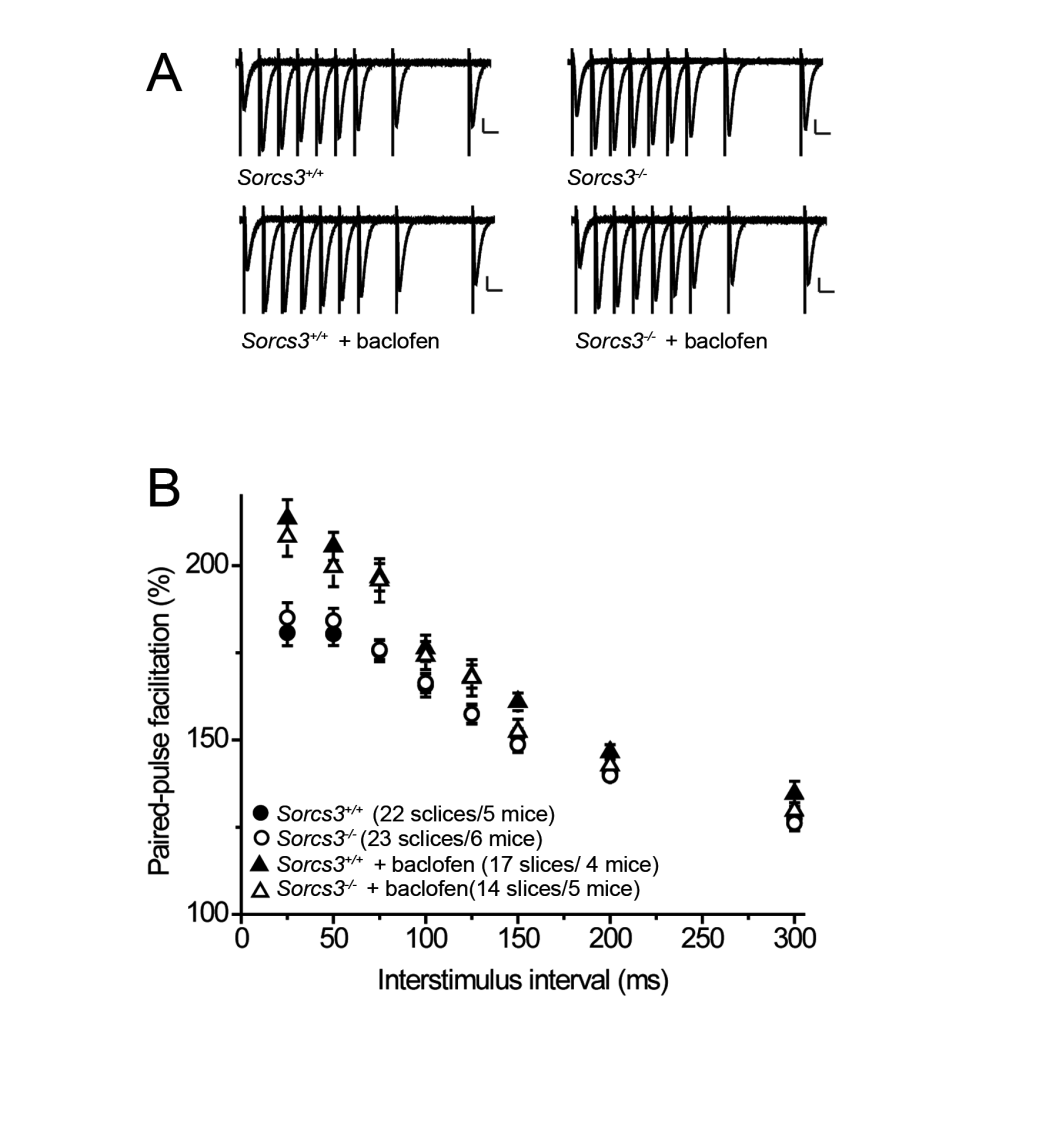
Normal paired-pulse facilitation in SORCS3-deficient mice. (**A**) Representative traces of fEPSPs after paired pulses with increasing interpulse intervals are depicted for the indicated genotypes and conditions. Scale bars: 0.3 mV/20 ms. (**B**) Paired-pulse facilitation (PPF) was calculated as the ratio of the second fEPSP slope to the first fEPSP slope and plotted at different interstimulus intervals. No significant differences (p>0.05) at any interstimulus intervals were seen comparing *Sorcs3* ^*+/+*^ and *Sorcs3* ^*-/-*^ mice. Application of the GABA_B_ receptor agonist baclofen increased the PPF ratio equally in mice of both genotypes. Note that y-axis starts at 100%. Data are given as mean ± SEM.

To exclude possible presynaptic effects of SORCS3 deficiency, we tested paired-pulse facilitation (PPF), a form of short-term synaptic plasticity [23]. In PPF, postsynaptic field potentials evoked by extracellular stimulation are increased at the second pulse due to enhanced release of synaptic vesicles from the presynaptic terminal [24]. As shown in Figure 5, there was no difference in PPF in mutant as compared to wild type mice. The GABA_B_ receptor agonist baclofen reduces the release probability primarily by inhibiting calcium channels, leading to a larger pool of release-ready vesicles for the second pulse [25]. Therefore, PPF is increased. Application of baclofen did not differently affect PPF in SORCS3-deficient mice compared with control animals, indicating intact presynaptic GABA_B_ receptor signaling cascades (Figure 5).

### Impaired learning and fear memory in mice lacking SORCS3

**Figure 6:**
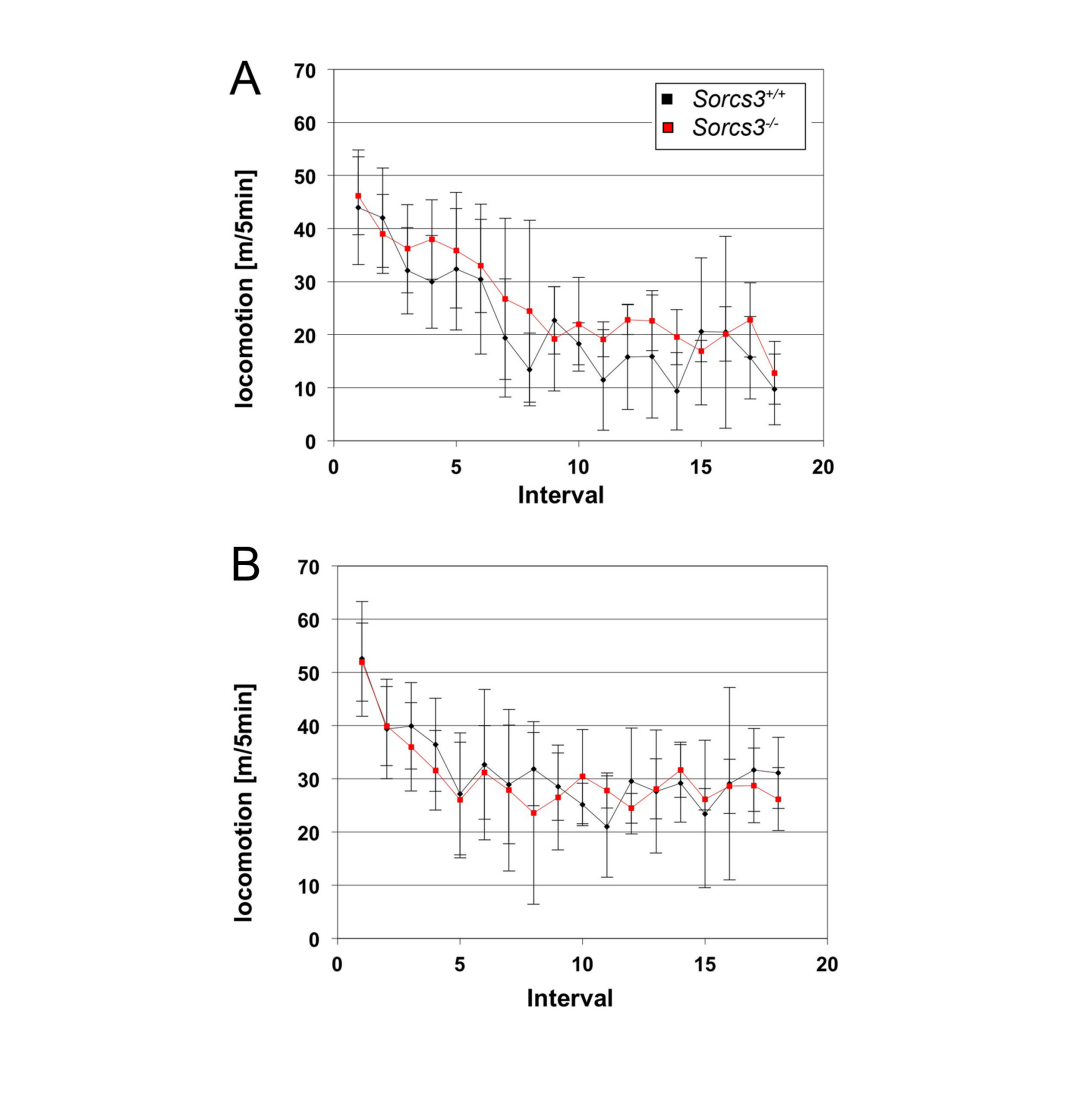
Normal behavior of SORCS3-deficient mice in the open field paradigm. Locomotion of *Sorcs3* ^*+/+*^ and *Sorcs3* ^*-/-*^ mice in an open field test run for 90 min on day 1 (panel A) and day 3 (panel B) of the test (n=3 per genotype). The distance traveled over a period of 5 minutes was averaged for each time interval (mean ± SD).

**Figure 7:**
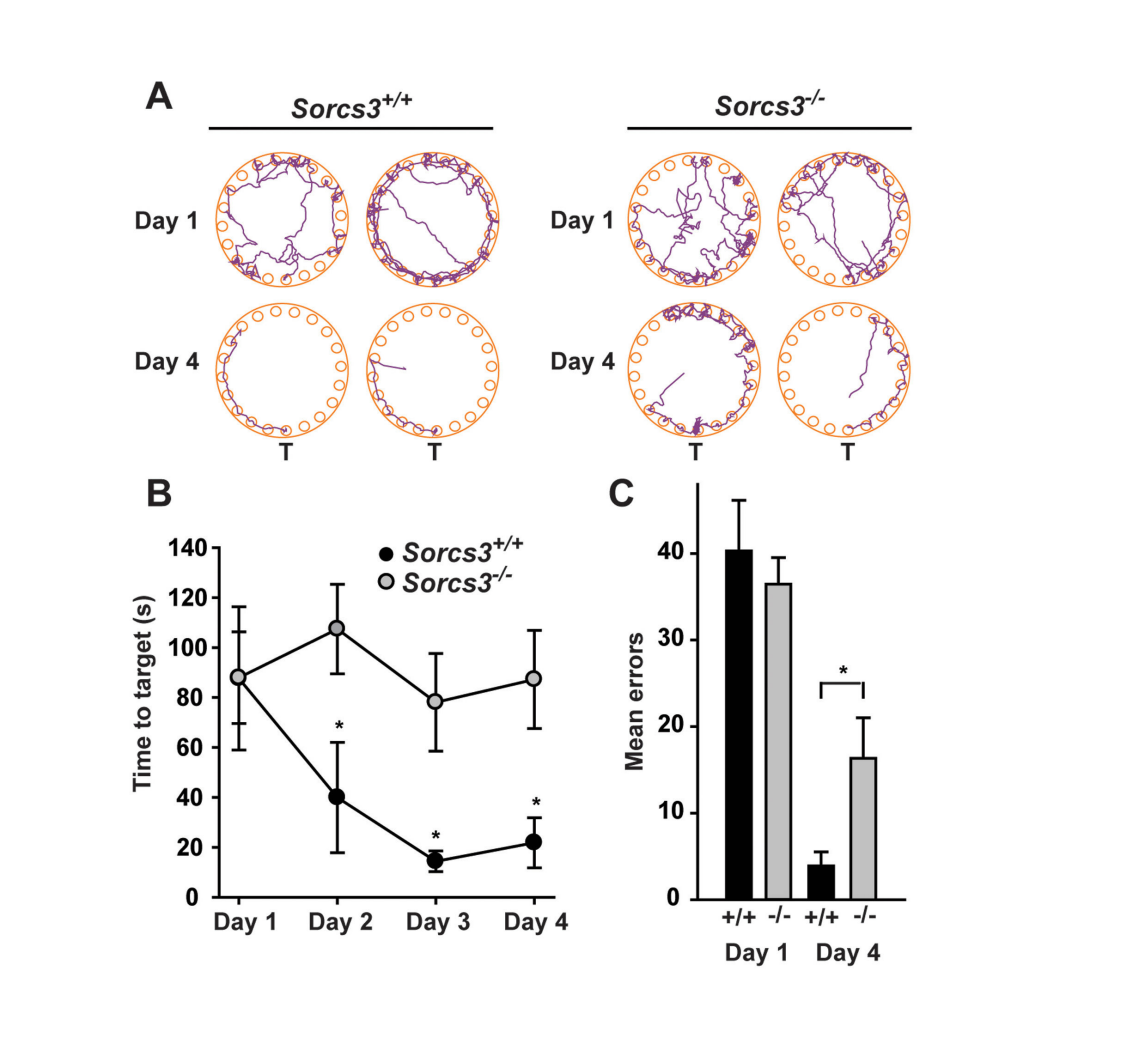
*Sorcs3* ^*-/-*^ mice display defects in spatial learning and memory in the Barnes maze. (**A**) Representative track plots illustrate the deficiency of individual *Sorcs3* ^*-/-*^ animals to locate the target hole on day 4 of the trial as compared to *Sorcs3* ^*+/+*^ controls. (**B**) Wild type mice (*Sorcs3* ^*+/+*^, n=7) display a steep learning curve as illustrated by the decreasing time required to enter the target hole during the four test days. In contrast, *Sorcs3* ^*-/-*^ mice (n=15) fail to acquire spatial memory. Statistical analysis was performed by a two-way ANOVA documenting a significant effect of genotype on learning performance: F=5.11, *p*<0.5. Subsequently, *post* *hoc* analyses were performed at individual time points using the two-tailed Student’s *t*-test for independent samples at each time point (*p<0.05). (**C**) *Sorcs3* ^*-/-*^ mice show significantly more nose poke errors on the fourth test day compared to wild-type controls. Two-tailed Student’s *t*-test was used for testing independent samples at day 4 (*p<0.05).

To explore the consequences of altered synaptic plasticity for learning and memory, we subjected SORCS3 null mice to a number of behavioral tests. In the open-field test, *Sorcs3* ^*-/-*^ mice failed to show any difference in locomotion compared to controls (Figure 6). However, when tested for spatial learning and memory using the Barnes maze (see methods for details), SORCS3-deficient animals performed significantly poorer than control animals with an obvious inability to learn the location of the escape target hole over a 4-day test period (Figure 7A and B) and a significantly increased number of nose poke errors on the fourth test day (Figure 7C).

**Figure 8:**
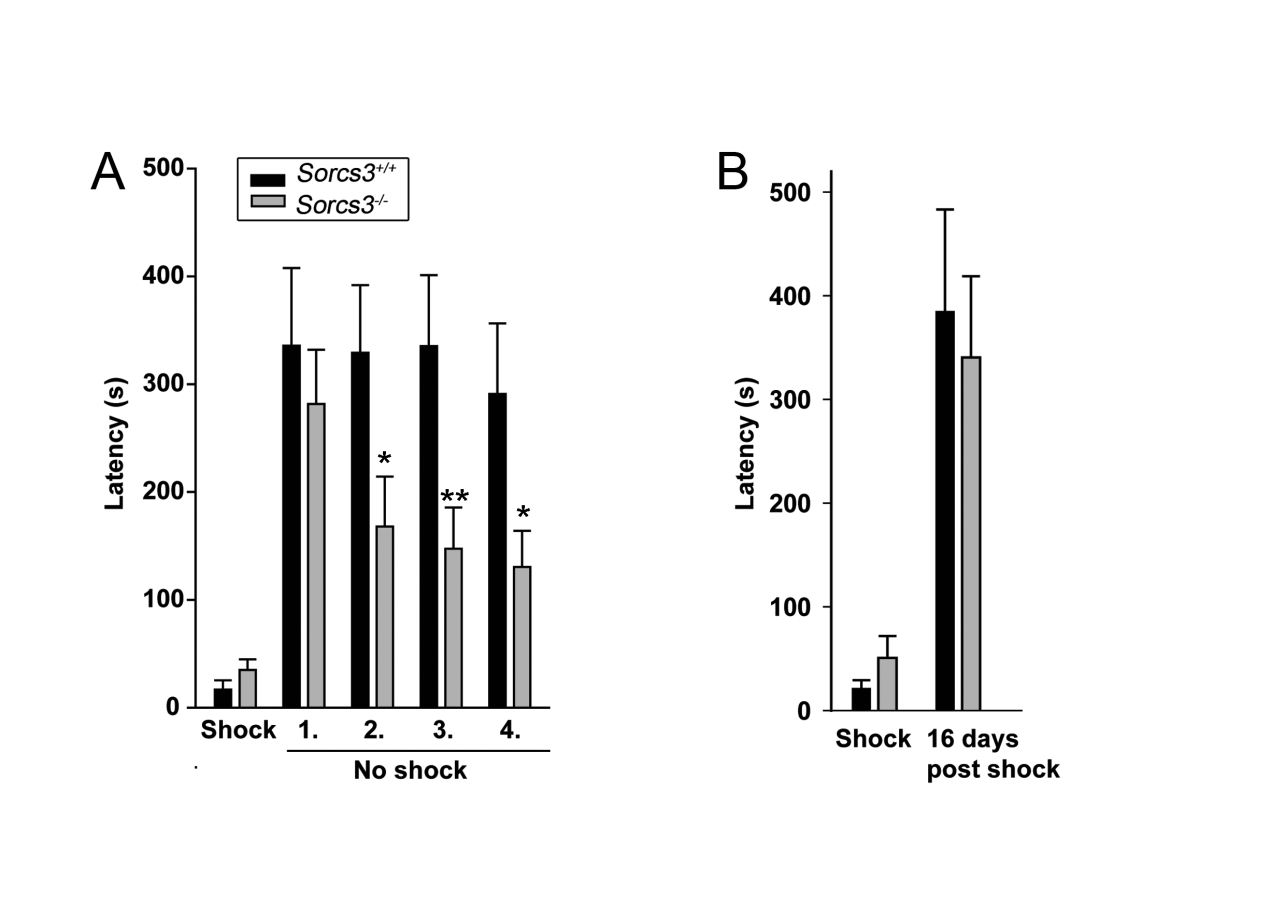
Lack of SORCS3 results in increased fear extinction in mice. (**A**) Mice were exposed to an inhibitory avoidance experiment as described in the method section. SORCS3-deficient mice display a more rapid extinction of fear over four consecutive days albeit at an intact acquisition of fear memory (shock). In contrast, wild type mice show no reduction in fear memory during the course of the experiment. Statistical analysis was performed by two-way ANOVA documenting a significant effect of genotype on learning performance: F=5.57, *p*<0.5. Subsequently, *post* *hoc* analyses were performed at individual time points using the two-tailed Student’s *t*-test for independent samples (*p<0.05, **p<0.01). (**B**) SORCS3-deficient and wild type mice display similar remote fear memory 16 days after the foot shock.

Fear memory is another form of long-lasting memory involving the hippocampus. Accordingly, we applied an inhibitory avoidance test to investigate fear memory in our mouse model. This setup takes advantage of the natural preference of mice for the dark and consists of a brightly lit room and a dark room separated by a guillotine door. On the training day, mice are placed in the bright room. When entering the dark room, the animals receive an electric foot shock. Twenty-four hours later, the mice are returned to the bright room and their latency to enter the dark area is recorded as an indicator of memory of the adverse event. Both wild type and receptor-deficient mice displayed a marked increase in their latencies to enter the dark room 24 h post shock, showing intact and similar acquisition of fear memory in both genotypes (Figure 8A, day 1). Subsequently, extinction of fear was tested by returning the animals once every 24 h into the setup and by monitoring their latency to cross into the dark room for a total of 4 consecutive days. As seen in Figure 8A, *Sorcs3* ^*-/-*^ mice displayed a significantly accelerated extinction of short-term fear memory as indicated by their willingness to enter the dark room faster than control animals on days 2 through 4 of the trial. This behavioral abnormality was not due to increased forgetting over time as the remote fear memory retrieval of SORCS3-deficient mice was identical to that of wild type controls when shocked and returned to the setup once after 16 days (Figure 8B).

### SORCS3 affects localization of PICK1 at the postsynapse

Based on the alterations seen in SORCS3-deficient mice, SORCS3 seems to play a role in weakening of synaptic strength. Possibly, up-regulation of *Sorcs3* by synaptic activity serves as a mechanism to balance synaptic hyperactivity [16]. One hypothesis concerning the molecular mechanism of SORCS3 action is involvement of this receptor in intracellular protein trafficking. As NMDA receptor-dependent and mGlu receptor-dependent LTD are lost in mutant mice, SORCS3 ought to act in a fundamental (trafficking) mechanism common to both forms of LTD (Figure 4).

**Figure 9:**
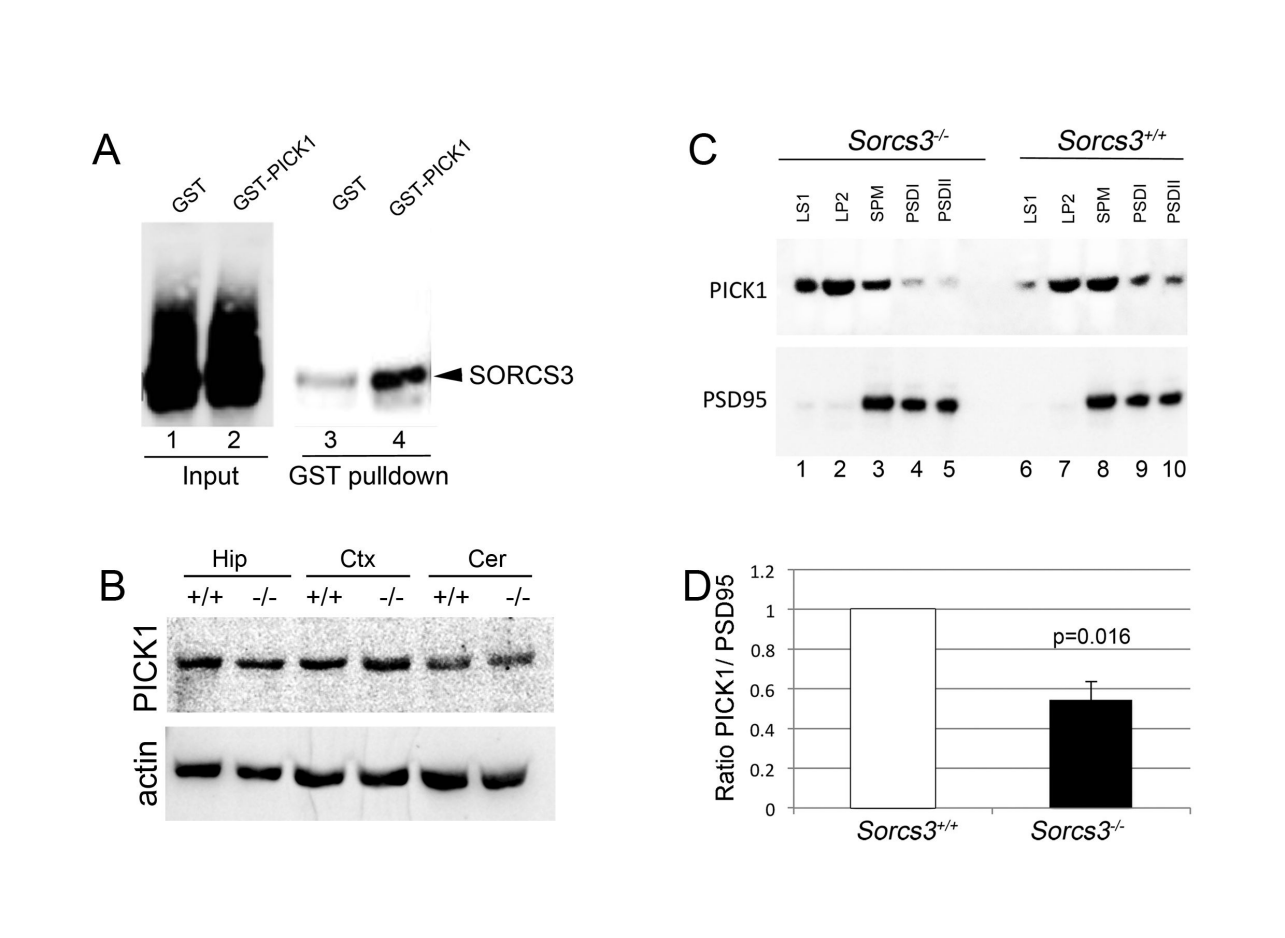
SORCS3 interacts with PICK1 and affects its localization at the post-synaptic density. (**A**) Lysates of HEK293 cells stably expressing SORCS3 were mixed with recombinant GST or GST-PICK1 as described in the method section. Pull-down experiments with glutathione sepharose beads (lanes 3-4) recovered SORCS3 from lysates containing GST-PICK1 (lane 4) but to a significantly lesser extent from GST-containing lysates (lane 3). Panel Input (lanes 1-2) documents the presence of SORCS3 in both samples prior to pull-down. (**B**) Immunodetection of PICK1 on extracts of hippocampus (Hip), cortex (Ctx), and cerebellum (Cer) documents equal levels of the protein in wild type (+/+) and SORCS3-deficient mice (-/-). (**C**) Representative Western blot analysis of synaptosomal preparations of wild type and *Sorcs3* ^*-/-*^ mouse hippocampi for PICK1 documents reduces levels of PICK1 in the SORCS3-deficient PSD as compared to the wild type control. Fractions were further probed against PSD95 as a control for accuracy of fractionation (absent in the synaptic vesicle preparation, LP2) and of equal loading. LS1, input supernatant for synaptic vesicle fraction; LP2, synaptic vesicle preparation; SPM, synaptic plasma membrane; PSDI and PSDII, postsynaptic densities. (**D**) Densitometric measurement of PICK1 levels in PSDI and PSDII fractions (as shown in panel B) from four independent experiments (13 mice per genotype). Intensities for PICK1 from PSDI and PSDII were combined and normalized against PSD95 for each experiment.

A cellular mechanism underlying both NMDA receptor-dependent and -independent forms of LTD is the endocytosis of AMPA receptors from the postsynaptic membranes, processes controlled by interaction of the glutamate receptors with anchoring proteins (reviewed in 26). PSD95 is mainly responsible for tethering of AMPA receptors to the synaptic membrane, with evidence showing that synaptic depression requires dephosphorylation of serine-295 of PSD95 [27]. On the other hand, PICK1 (protein interacting with C-kinase 1) emerges as a sorting adaptor implicated in removal of AMPA receptors from the synaptic cell surface during LTD [28,29,30,31,32]. Especially, PICK1 has been shown to be essential for the clustering and intracellular retention of the AMPA receptors, a mechanism that underlies the expression phase of the LTD [32,33]. Accordingly, we tested whether PICK1 may represent an adaptor protein also interacting with the cytoplasmic domain of SORCS3. Interaction of PICK1 with SORCS3 was confirmed by pull-down of the receptor from transiently transfected HEK293 cells using a recombinant GST-PICK1 fusion protein (Figure 9A). Total levels of PICK1 were unchanged in hippocampus, cortex or cerebellum of SORCS3-deficient mice (Figure 9B). However, subcellular fractionation studies documenting reduced levels of PICK1 in the PSD of SORCS3-deficient compared to wild type hippocampi. This observation supported the functional significance of PICK1 and SORCS3 interaction for adaptor sorting *in vivo* (Figure 9C and D).

## Discussion

VPS10P domain receptors emerge as central regulators of neuronal viability and function. Their mode of action commonly involves the ability to interact with cytosolic adaptors to sort target proteins, such as Trk receptors [9] or APP [11,15,34,35]. Studies presented herein support this notion by elucidating a hitherto unknown function for the orphan receptor SORCS3 in control of LTD, an activity potentially involving PICK1-dependent sorting processes as the postsynapse.

SORCS3 is a member of the VPS10P domain receptor gene family that shows the most restricted pattern of expression, with the main site of expression being the hippocampus. The receptor is also unique in as much as it is the only VPS10P domain receptor to harbor a PDZ domain binding motif (X-S/T-X-V), arguing for a specific role of this protein at the postsynapse of hippocampal neurons not shared by the other receptors. A role for SORCS3 in synaptic activity was now confirmed by documenting distinct defects in synaptic plasticity related to LTD in mice genetically deficient for this receptor. Postsynaptic dysfunctions coincide with distinct behavioral anomalies in affected animals including defect in spatial learning and memory, and in enhanced extinction of contextual fear.

Spatial learning as tested in the Barnes maze requires hippocampal function. Also, modulation of fear through repeated exposure to the context of an adverse event is controlled by hippocampal activities [36], providing a behavioral correlate for the alteration in synaptic plasticity seen in the hippocampus of *Sorcs3* ^*-/-*^ mice. Thus, applying antagonists of NMDA receptor subunits to specifically erase LTP versus LTD, blockade of LTD (but not of LTP) in the CA1 region of the hippocampus of rats resulted in impaired consolidation of spatial memory in the Morris water maze [37]. These data are in line with studies in knockout mouse models wherein selective loss of LTD [38,39] but not of LTP [40,41] affected spatial learning. Our data in SORCS3-deficient mice support the concept that LTD in the CA1 region is of particular importance for establishing long-term spatial memory, a function that apparently involves SORCS3 activity.

LTD is also implicated in other forms of learning such in the extinction of aversive memories. For example, mice overexpressing Rap2, a Ras-related GTPase at the synapse, display normal LTP but enhanced LTD, which, in turns, results in impaired extinction of contextual fear [42]. In line with such an inverse correlation, loss of LTD in *Sorcs3* ^*-/-*^ animals coincides with accelerated inhibition of contextual fear memory (Figure 8). Several psychiatric disorders such as major depression or posttraumatic stress disorder syndrome are caused in part by a failure to erase aversive memory. Given the functional implication of *Sorcs3* in modulation of fear memory in mice, this gene may represent a novel risk gene for anxiety-related disorders in humans. Although highly speculative at present, one genetic study reported a *de novo* duplication of the chromosomal region 10q23 encoding *SORCS1* and *SORCS3* in an individual with bipolar disorder [43]. Intriguingly, bipolar disorder is known to share high comorbidity with posttraumatic stress disorder syndrome [44].

What may be the molecular mechanism of SORCS3 action in hippocampal neurons? Based on analogy to other VPS10P domain receptors, sorting of target proteins at the postsynapse seems a plausible hypothesis. This model is supported by the ability of the receptor to interact *in vitro* with PSD95 and PICK1, adaptors implicated in glutamate receptor localization. In a simple scenario, our data suggest a model whereby SORCS3 interacts with PICK1 to modulate AMPA receptor sorting at the PSD. Inhibitory peptides or antibodies blocking the interaction of PICK1 with GluR2/3 attenuate LTD induction in cerebellar Purkinje cells in culture [45]. Furthermore, inactivation of PICK1 by gene ablation [28,30,46] or by pharmacological intervention [29] impairs LTD in the cerebellum as well as LTP and LTD in the CA1 region of the hippocampus. Because LTD but not LTP are affected by loss of SORCS3, the interaction of PICK1 with this receptor seems relevant for removal of AMPA receptors from but not for targeting to the postsynaptic membrane compartment. Total levels of PICK1 are not changed in the hippocampus of *Sorcs3* ^*-/-*^ mice (Figure 9B). However, we observed a distinct loss of the adaptor protein from the PSD fraction, arguing for a redistribution of the protein between subcellular compartments in the absence of SORCS3 (Figure 9C and D). So far we failed to detect any major changes in levels of AMPA receptor subunits in the PSD of SORCS3-deficient hippocampi using subcellular fractionation studies (Figure 2E). However, more drastic interventions, such as induction of synaptic activity by kainic acid injection, may be required to better appreciate altered localization of AMPA receptors in SORCS3-deficient hippocampal neurons.

In conclusion, we have identified a hitherto unknown function for SORCS3 as regulator of LTD, further underscoring the central roles played by VPS10P domain receptors in control of neuronal activities. Further elucidation of this pathway will help in understanding the molecular mechanisms underlying repression of synaptic activity and how alterations in components of this pathway may contribute to anxiety-related disorders in humans.
